# Socioeconomic inequalities in the risk of SARS-CoV-2 infection – First results from an analysis of surveillance data from Germany

**DOI:** 10.25646/7057

**Published:** 2020-10-09

**Authors:** Benjamin Wachtler, Niels Michalski, Enno Nowossadeck, Michaela Diercke, Morten Wahrendorf, Claudia Santos-Hövener, Thomas Lampert, Jens Hoebel

**Affiliations:** 1 Robert Koch Institute, Berlin Department of Epidemiology and Health Monitoring; 2 Robert Koch Institute, Berlin Department of Infectious Disease Epidemiology; 3 University of Düsseldorf Medical Faculty, Institute of Medical Sociology, Centre for Health and Society

**Keywords:** COVID-19, SARS-COV-2, HEALTH INEQUALITIES, REGIONAL SOCIOECONOMIC DEPRIVATION

## Abstract

Experiences with acute respiratory diseases which caused virus epidemics in the past and initial findings in the research literature on the current COVID-19 pandemic suggest a higher SARS-CoV-2 infection risk for socioeconomically disadvantaged populations. Nevertheless, further research on such a potential association between socioeconomic status and SARS-CoV-2 incidence in Germany is required. This article reports on the results of a first Germany-wide analysis of COVID-19 surveillance data to which an area-level index of socioeconomic deprivation was linked. The analysis included 186,839 laboratory-confirmed COVID-19 cases, the data of which was transferred to the Robert Koch Institute by 16 June 2020, 00:00. During the early stage of the epidemic up to mid-April, the data show a socioeconomic gradient with higher incidence in less deprived regions of Germany. Over the course of the epidemic, however, this gradient becomes less measurable and finally reverses in south Germany, the region hardest hit by the epidemic, to the greater detriment of the more deprived regions. These results highlight the need to continue monitoring social epidemiological patterns in COVID-19 and analysing the underlying causes to detect dynamics and trends early on and countering a potential exacerbation of health inequalities.

## 1. Introduction

First detected in Wuhan, China, the SARS-CoV-2 (Severe Acute Respiratory Syndrome Coronavirus 2) [1] coronavirus has continued to spread and poses a considerable challenge to societies globally. During the early stages of the pandemic, it was repeatedly claimed that the virus would affect all people more or less equally [2], yet international publications meanwhile indicate that socioeconomically disadvantaged populations run a higher SARS-CoV-2 infection risk (see Focus article Socioeconomic inequalities and COVID-19 – A review of the current international literature in this issue of the Journal of Health Monitoring) [3–6]. Similar differences in infection risks by socioeconomic status have already been described for the 2009 and 1918 influenza pandemics [5, 7, 8] and for the viral pathogens causing seasonal epidemics of acute respiratory diseases [9–11].

Socioeconomic inequalities in infection and disease risks could be owed to differences in virus exposure and susceptibility [8, 12]. Most likely, these are to a great degree related to differences in living and working conditions, behaviour and psychosocial factors [8]. Being able to stay away from work without severe financial loss is potentially a considerable factor for the probability of transmission [8, 13, 14]. Being able to work from home, however, is an option mainly open to more highly qualified professions and high income earners [15]. Housing conditions, too, are likely to influence a person’s infection risk. During the 2009 influenza pandemic, patients in the US who lived in crowded conditions were more likely to suffer a severe case of their influenza infection [16]. First studies, also from the US, now report such an association between crowded living conditions and a higher infection risk for SARS-CoV-2 as well [17, 18]. Housing in Germany, in particular in the large cities, is also not equally distributed with low income groups having less space [19].

In addition to the direct living and work conditions, psychosocial and behavioural factors could also play a role for the socioeconomic inequalities in SARS-CoV-2 susceptibility [20]. An experimental study, for example, showed that people who perceive themselves as socially disadvantaged are at a greater risk of developing a symptomatic infection after exposure to a cold virus [20, 21]. Behavioural factors, too, such as physical activity or dietary habits, can influence susceptibility and are also distributed unequally [20]. Based on the differences described in terms of exposure and susceptibility, it seems plausible to assume that socioeconomically disadvantaged populations have a higher risk for SARS-CoV-2 infection and developing COVID-19.

While international findings in particular from the US and the UK indicate that the distribution of SARS-CoV-2 infections is skewed towards people in low socioeconomic groups (see additional Focus article in this issue of the Journal of Health Monitoring) [4, 5], very little research into such an association for Germany and other European countries has so far been conducted. This article therefore seeks to answer the question as to whether and at what early stages of the epidemic in Germany, did socioeconomic inequalities play out in SARS-CoV-2 infection risks and whether changes regarding such an effect occurred over the course of the first months of the COVID-19 epidemic in Germany. Such analyses are suited to monitoring the trends of socioeconomic inequalities in infections and can contribute to identifying further at-risk groups for a SARS-CoV-2 infection and potentially show options for targeted infection protection measures.

## 2. Methods

### 2.1 Data

The analysis is based on the official surveillance data on notifiable infectious diseases held by the Robert Koch Institute (RKI). Germany’s Protection against Infection Act (IfSG) mandates the notification of confirmed SARS-CoV-2 diagnoses, suspected COVID-19 infections, disease development and deaths. Physicians and laboratories are responsible for reporting cases. They report to local health authorities, who report to the federal state level authorities, who then report figures to the RKI. The analyses considered laboratory-confirmed cases up to 15 June 2020 that contained information on the notification date (the date on which a case was reported to and electronically recorded at local health authorities), sex, age and district of the responsible health authority (data as at: 16 June 2020, 00:00).

### 2.2 Socioeconomic deprivation

To analyse socioeconomic inequalities in the COVID-19 incidence, the data reported to the RKI was combined with the German Index of Socioeconomic Deprivation (GISD) [22, 23]. GISD measures the degrees of socioeconomic deprivation of regional populations in Germany and for this analysis serves as a proxy for socioeconomic status. This regional measure was used because the surveillance data does not provide individual socioeconomic data such as occupational position or highest educational qualification of those infected. GISD was developed by the RKI specifically for epidemiological research and health reporting in Germany and is available for a number of spatial levels. For this analysis, GISD was used at the level of the 401 administrative districts in Germany, as the district level is the most finely grained spatial unit that can be analysed with the RKI’s German surveillance data.

The GISD is a multi-dimensional index of aggregated area-based indicators for the three core dimensions of socioeconomic status – education, employment and income. The core data stem from the INKAR (indicators and maps on spatial and urban development) database of Germany’s Federal Institute for Research on Building, Urban Affairs and Spatial Development [24]. The educational dimension covers data on the regional proportion of the workforce with a university or university of applied science degree and the proportion of school leavers without a school leaving certificate. During the first revision of the index, this data was supplemented with the proportion of the workforce not holding a professional qualification and the quota of school leavers with a certificate qualifying them to study at university. The employment dimension uses data on regional levels of unemployment (unemployed persons per 1,000 inhabitants of active age), the average gross monthly salary and the employment ratio (number of insured employed persons per 100 inhabitants of active age). The income dimension uses the regional average household income (disposable income of private households), debtor rates (private debtors per 100 inhabitants) and the average revenue from income tax per inhabitant. Education, employment and income are allocated the same weight in the total index score, which ranges from 0 (lowest level of deprivation) to 1 (highest level of deprivation). For the analysis, districts were separated into five equally large groups (quintiles), where the first quintile represents the 20% least and the fifth quintile the 20% most deprived districts in Germany.

### 2.3 Statistical analysis

For statistical analysis, the number of notified cases was related to the population [25] and the cumulative incidence (number of cases per 100,000 inhabitants) since the start of the epidemic in Germany was calculated. To identify socioeconomic inequalities in incidence, these rates were calculated for each quintile of socioeconomic deprivation. To recognise trend dynamics, a number of different periods was analysed, beginning with the phase up to 15 March 2020 and then for each month up to 15 June. Cases were assigned to a phase based on the date of notification.

As the deprivation quintiles differ in their population’s age composition and different age groups face different COVID-19 risks [26], direct age standardisation was used to calculate age-standardised incidence rates. The revised 2013 European Standard Population was used as standard population [27]. Incidence rates can then be directly compared between deprivation quintiles because standardisation adjusts for differences in age structures. To identify potential sex differences, all analyses were conducted separately for women and men.

## 3. Results

**Figure 1:**
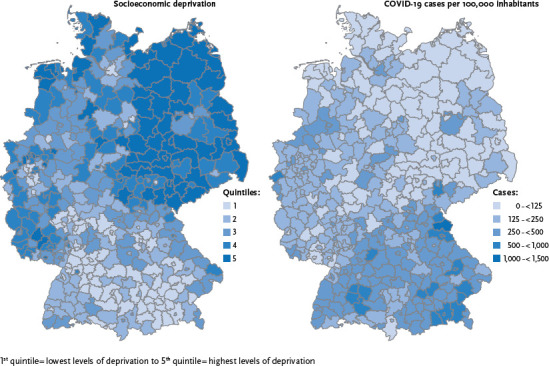
Regional distribution of socioeconomic deprivation and the age-standardised COVID-19 incidence at the district level in Germany Source: Kroll et al. 2017 [22, 23], RKI surveillance data (as at 16 June 2020, 00:00)

By 16 June 2020, 00:00, the RKI had received data on 186,839 laboratory-confirmed cases of COVID-19. Relative to the population, this was a cumulative incidence of 231 cases per 100,000 women and 218 cases per 100,000 men. Figure 1 shows regional levels of socioeconomic deprivation and the age-standardised cumulative COVID-19 incidence for Germany’s 401 districts. It appears that for the period analysed, the southern regions of Germany, and therefore in particular districts in the states of Bavaria and Baden-Wuerttemberg, were, geographically, the areas in Germany that – while the least deprived – were hardest hit by the COVID-19 epidemic.

Table 1 shows the age-standardised incidence rates by quintiles of socioeconomic deprivation. Both sexes present a clear socioeconomic gradient with a higher cumulative COVID-19 incidence in less deprived regions. After age standardisation, the cumulative incidence is 2.4 times (women) and 2.7 times (men) higher in the lowest than in the highest deprivation quintile.

**Figure 2:**
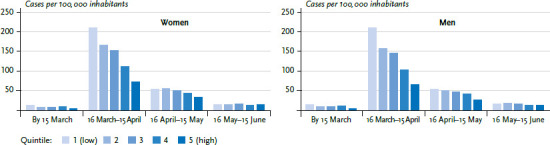
Age-standardised COVID-19 incidence in Germany by socioeconomic deprivation and notification period Source: RKI surveillance data (as at 16 June 2020, 00:00)

Figure 2 shows the results for Germany by quintiles of deprivation (coloured bars) at points in time (x-axis). Such a presentation shows the dynamics of the socioeconomic gradient in COVID-19 incidence over the observation period. A socioeconomic gradient with higher incidence rates in less deprived districts is found in particular up to mid-April, i.e. during the early phase of the epidemic in Germany. From mid-April, the number of cases dropped considerably, as did the role played by socioeconomic differences. Over the course of the epidemic, then, the regional socioeconomic gradient visibly flattened. From mid-May, our Germany-wide analysis could no longer detect a gradient with a higher COVID-19 incidence in less deprived districts.

**Figure 3:**
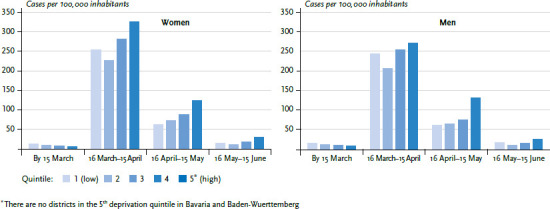
Age-standardised COVID-19 incidence in south Germany (Bavaria and Baden-Wuerttemberg) by socioeconomic deprivation and notification period Source: RKI surveillance data (as at 16 June 2020, 00:00)

In Germany’s southern regions of Bavaria and Baden-Wuerttemberg, where considerably more COVID-19 cases were notified until mid-April 2020 than in most other regions, the socioeconomic gradient reverses over time. Whereas during the initial phases of the epidemic until mid-March 2020, the COVID-19 incidence was highest in the least deprived districts, from mid-April on the gradient reverses and figures are now higher in districts where deprivation is higher (Figure 3). This effect is observed for both women and men. The figures indicate that during the later stages of the epidemic in south Germany, people were hit harder by the epidemic when they lived in socioeconomically more deprived districts.

## 4. Discussion

This first empirical analysis of national surveillance data on notified COVID-19 cases in Germany which have been combined with an index of regional socioeconomic deprivation (GISD) show that until mid-April 2020 the standardised incidence rate was higher in less deprived districts. From mid-May, Germany-wide analysis could no longer detect this socioeconomic gradient. Overall, considerable regional socioeconomic inequalities in incidence were observed, with the two southern federal states of Bavaria and Baden-Wuerttemberg bearing the main burden of infections. For this southern part of Germany, our analyses show that with an overall decreasing incidence rate, the initially visible socioeconomic gradient with higher rates in less deprived districts begins to reverse. From mid-April, higher incidence rates are observed in the more socioeconomically deprived districts. These findings are broadly in line with the results of the only published, yet not peer-reviewed study, from Germany during the observation period [28], which had found an initially higher infection risk in high socioeconomic status regions, which then reversed to a greater risk in regions of low socioeconomic status. Our study confirms and expands these findings by showing not only the shifts in socioeconomic inequalities over the course of the pandemic, but also the regionally differentiated picture of these changes.

To put the results presented here on the potentially changing patterns of socioeconomic inequality over the course of the outbreak in the incidence of COVID-19 in context, we must look at the course the epidemic took in Germany up to this point. In Germany, the COVID-19 epidemic began with the first documented case in the Starnberg district at the end of January 2020 [29, 30]. It was directly related to the initial outbreak in Wuhan, China, and was the first proven case of human-to-human transmission outside of Asia. Later, trips to other European countries and, in particular, people who brought SARS-CoV-2 back with them from skiing trips to the Alps seem to have played an important role. Symbolically this is represented by Austria’s Ischgl ski resort in Tirol [31]. However, it must be assumed that people returning from other parts of Austria and northern Italy also brought the virus in. Certain facts appear to indicate that the early phases of the pandemic were characterised both globally and in Germany by individual events that lead to an increased transmission of the virus. Two preprint studies have concluded that around 80% of transmissions can be tracked to 10% of the infected population [32, 33]. A further preprint study indicates that 20% of the infected population are responsible for 80% of all transmissions [34]. In Germany, several districts with such events have become well documented and these outbreaks were (or are still being) scientifically studied during the weeks and months thereafter: Heinsberg [35], Tirschenreuth [36], Hohenlohe and Rosenheim [37]. These districts remain among those with the highest cumulative incidence rates in Germany [26]. The factors influencing the subsequent development of regional incidence have been analysed for example by a study in Saxony-Anhalt’s rural Wittenberg district [38]. When compared to other districts, the relatively low number of cases facilitated the documentation of the spread of the disease from a cluster in the town of Jessen, which could be tracked to people who had returned from Austria [38]. The study concluded that the virus had then subsequently spread through family and/or household members, colleagues at work, as well as through friends and acquaintances. The factors which had encouraged and inhibited the (trans)regional spread of the virus have also been analysed by a study of the German Institute for Economic Research (DIW). For the period before the lock-down, the findings indicate that greater population density and bad weather lead to an increased spread of SARS-CoV2, as both factors increased the contact probability. However, according to the study, commuters are a more important factor to take into account. If two districts are highly linked through commuters, this contributes to the spread of the virus [39]. Well-connected districts therefore refer to districts where a large proportion of the workforce commutes to another district.

The observed dynamics for the initial stages of the outbreak in Germany make it seem plausible that, during the early stages, regions with low levels of socioeconomic deprivation and people of relatively high socioeconomic status were hit harder because of the type of travelling seen – in particular skiing trips – as well as participation in social events requiring certain financial means. The importance of commuter travel during this phase also makes it plausible that, during this phase, districts with lower levels of deprivation and a higher level of economic activity were affected more. Moreover, proximity to the first risk areas in Europe, holiday-making habits and that the outbreak coincided with social events such as carnival and other celebrations could have also played a role.

However, the results of our analysis of German surveillance data on notifiable infectious disease also indicate that when the chain of human-to-human transmission persists, other social and/or socioeconomic factors potentially begin to play a key role and translate into a greater risk for people in low socioeconomic groups and these effects subsequently have the potential to exacerbate pre-existing health inequalities. Working and living conditions probably play an increasingly important role for SARS-CoV-2 infection risks here. Publications from the US and the UK, for example, show that crowded housing conditions can be related to an increased infection risk [17, 18] and Public Health England has reported that SARS-CoV-2 incidence is higher for staff in certain sectors such as health care, retail, hotels and restaurants or security [40, 41]. Even though these results from other countries are not directly comparable due to the differences in social systems and workplace health and safety regulations, these findings and our empirical analyses do indicate that in Germany, too, we should anticipate seeing people in low socioeconomic groups being more affected by COVID-19 throughout the epidemic.

The increased number of cases that have been registered since the end of April in meat plants, among labour migrants in agricultural jobs, as well as in refugee accommodation appear to confirm these assumptions although the inter-sectional marginalisation and discrimination these groups suffer with regard to working and living conditions as well as healthcare access presumably play key roles. How these infection clusters will play out for socioeconomic inequality requires further analysis. In Germany, the Competence Network Public Health COVID-19, an initiative of members from 25 associations with expertise in the public health fields, is working on the on-going interdisciplinary analysis of these questions [4, 42–43].

This study is the first Germany-wide analysis of COVID-19 surveillance data in combination with an area-based index of socioeconomic deprivation. Age standardisation enables direct comparisons between more and less deprived regions to be made because regional differences in age structure have been adjusted for. Therefore, the analysis can provide important insights regarding the development of the epidemic as well as a starting point for the future monitoring of socioeconomic inequalities in COVID-19 during the pandemic. However, this study also has certain weaknesses. The GISD applied is based on 2014 data and changes may have taken place since in the underlying regional indicators, even though regional levels of socioeconomic deprivation generally hardly tend to change in the period of a few years. Moreover, exclusively relying on area-level data means no direct conclusions on individual health differences can be made (ecological fallacy). Furthermore, these analyses cannot consider other factors that could influence the spread of the virus such as the density of the population and commuter links. Also, the analyses are based on aggregated socioeconomic data at district level and therefore cannot describe inequalities at other levels such as those of the individual or local communities. Hopefully these ecologic analyses can be supplemented in future by studies that use data at the level of individuals for Germany-wide analyses and can then provide greater insights into the underlying mechanisms that lead to socioeconomic inequalities in COVID-19. Nevertheless, this study has also shown the adequacy of the applied methodology in monitoring the development of socioeconomic inequalities in the incidence and possibly also mortality of COVID-19 over time by using the RKI’s national surveillance data on notifiable infectious diseases. The results should be viewed as a snapshot of a highly dynamic epidemic and will have to be followed up throughout the COVID-19 pandemic.

## Conclusion

The results of our analyses of COVID-19 surveillance data for Germany indicate that infection risks in Germany follow regional patterns of socioeconomic inequality. During the early stages of the epidemic in Germany, a socioeconomic gradient with higher incidence rates in socioeconomically privileged districts was observed and however began to shift over the continued course of the epidemic and reversed in the most affected southern regions of Germany after mid-April. Based on these findings, it must be apprehended that throughout the pandemic, socially disadvantaged people might suffer more from COVID-19 and that pre-existing health inequalities could become exacerbated. The possible trends urgently require further monitoring and should be supplemented by analyses of data at the individual level. The underlying mechanisms demand further analyses with a view to preventing an exacerbation of health inequalities using targeted measures and a targeted control of infections by protecting groups which are particularly vulnerable.

## Key statements

- There is insufficient research to understand the social epidemiological patterns of the COVID-19 epidemic in Germany.
- Socioeconomic inequalities in COVID-19 vary geographically and over time.
- Initially, German surveillance data for COVID-19 indicated a socioeconomic gradient with higher incidence rates in less deprived regions.
- Over the course of the epidemic in Germany, the regional socioeconomic gradient became considerably less defined and later reversed in south Germany.
- Monitoring the social epidemiological patterns of COVID-19 is necessary in order to counteract a potential exacerbation of health inequalities.

## Figures and Tables

**Table 1:** Age-standardised COVID-19 incidence in Germany by socioeconomic deprivation Source: RKI surveillance data (as at 16 June 2020, 00:00)

Socioeconomic deprivation	Women Cases per 100,000 inhabitants	Men Cases per 100,000 inhabitants
Quintile 1 – low	290	292
Quintile 2	242	234
Quintile 3	225	215
Quintile 4	176	167
Quintile 5 – high	121	108
